# Biological Membrane-Packed Mesenchymal Stem Cells Treat Acute Kidney Disease by Ameliorating Mitochondrial-Related Apoptosis

**DOI:** 10.1038/srep41136

**Published:** 2017-01-24

**Authors:** Xiaodong Geng, Quan Hong, Weiwei Wang, Wei Zheng, Ou Li, Guangyan Cai, Xiangmei Chen, Di Wu

**Affiliations:** 1 Department of Nephrology, PLA General Hospital, Institute of Nephrology, Beijing Key Laboratory of Kidney Disease, State Key Laboratory of Kidney Diseases, National Clinical Research Center for Kidney Diseases, 28 Fuxing Road, Beijing 100853, China; 2 Department of Thoracic Surgery, Peking Union Medical College Hospital, Chinese Academy of Medical Sciences and Peking Union Medical College, No. 1 Shuai Fu Yuan, Eastern District, Beijing 100730, China

## Abstract

The mortality of rhabdomyolysis-induced AKI remains high because no effective therapy exists. We investigated a new therapeutic method using MSCs. The aim of this study was to investigate the therapeutic potential and anti-apoptotic mechanisms of action of MSCs in the treatment of AKI induced by glycerol *in vivo* and *in vitro*. We used Duragen as a biological membrane to pack MSCs on the glycerol-injured renal tissue *in vivo*. The anti-apoptotic mechanism was investigated. *In vitro*, HK-2 cells were incubated with ferrous myoglobin and MSCs-conditioned medium, followed by cell proliferation and apoptosis assays. We founded that packing MSCs on the injured renal tissue preserved renal function, ameliorated renal tubular lesions, and reduced apoptosis in the mice with glycerol-induced AKI. The MSC-conditioned medium improved HK-2 cell viability and inhibited apoptosis. These effects were reversed by the PI3K inhibitor LY294002. Biological membrane packing of MSCs on the renal tissue has a therapeutic rescue function by inhibiting cell apoptosis *in vivo*. MSCs protect renal cells from apoptosis induced by myoglobin *in vitro*. We have thus demonstrated MSCs reduced rhabdomyolysis-associated renal injury and cell apoptosis by activating the PI3K/Akt pathway and inhibiting apoptosis.

Acute kidney injury (AKI) is an increasingly prevalent complex clinical disorder. Intrinsic AKI, which results from direct damage to the kidney, may arise in a number of ways, including drug/toxicant exposure or ischemia^1^. Rhabdomyolysis(RM) is massive breakdown of skeletal muscles and liberation of their content into the blood stream^2^. Myoglobin-induced renal toxicity plays a key role in rhabdomyolysis-associated kidney damage by increasing oxidative stress, inflammation, endothelial dysfunction, vasoconstriction, and apoptosis^3^.

Experimental AKI induced by glycerol injection is a well-established model of rhabdomyolysis^4^. It is characterized by intense cortical acute tubular necrosis and inflammatory cell infiltration^5^. Mesenchymal stem cells (MSCs) have been used as therapeutic agents for immunomodulation and tissue repair, which highlights their potential for recovery from kidney injury^6^. The mechanisms responsible for their protective roles most likely involve paracrine and endocrine effects, including mitogenic, anti-apoptotic and anti-inflammatory effects^7^. Several pathways are involved in glycerol-induced AKI. The phosphatidylinositol 3-kinase/v-aktmurinethymoma viral oncogene homolog(PI3K/Akt) pathway is an important signaling pathway for cell survival^8^. ROS is a by-product of the normal cellular metabolism; however, excessive amounts can cause detrimental effects. In particular, ROS is an important factor in the apoptosis pathway; increased ROS generation alters the mitochondrial membrane potential and damages the respiratory chain, which ultimately triggers the apoptotic process^9^,^10^. Apoptosis is an important phenomenon in glycerol–induced AKI. Anti-apoptotic proteins such as Bcl-xl and pro-apoptotic proteins such as Bad, caspase-3, and caspase-9 are involved in the process of apoptosis^11^. However, its underlying mechanisms in MSC-treated glycerol-induced AKI remain poorly understood.

Duragen is a collagen matrix synthesized from bovine Achilles tendon. It is a type of biological membrane(BM) that can be used to treat cerebrospinal fluid leaks^12^. Fibroblasts use the pores on the matrix to lay down endogenous collagen. By 6–8 weeks, the collagen matrix is resorbed and is integrated to the endogenous dura^13^. We used the biological membrane characteristics of Duragen to repair injured kidney tissue.

In this study, we used Duragen as a biological membrane to wrap MSCs; it was then packed on the injured kidney. This maneuver allowed us to permanently lodge MSCs into the injured kidney. First, we proved that this method resulted in the amelioration of rhabdomyolysis-induced AKI. Second, we used HK-2 human renal proximal tubule cells co-cultured with ferrous myoglobin*in vitro* and a glycerol-induced mouse AKI model *in vivo* to further understand the mechanisms responsible for the renoprotective effects of MSCs.

## Results

### Biological membrane-packed MSCs attenuate the histological and functional deterioration in glycerol-induced mouse kidney injury

**Figure 1:**
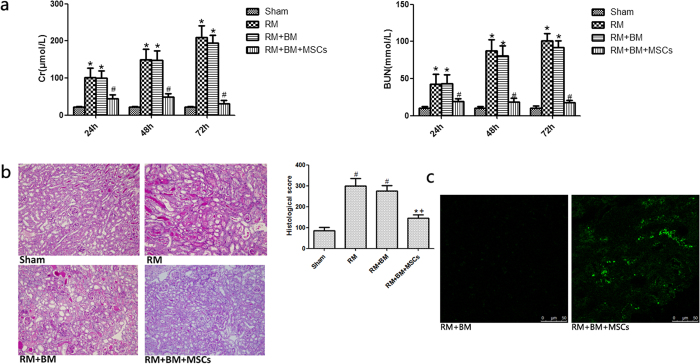
Biological membrane packing of Mesenchymal stem cells (MSCs) on the renal tissue ameliorate rhabdomyolysis (RM)-induced acute kidney injury (AKI). (**a**) Compared with sham mice, biological membrane packing of Mesenchymal stem cells (MSCs) on the renal tissue significantly reduced serum creatinine (SCr), blood urea nitrogen (BUN) levels 24, 48 and 72 hours after RM. *p < 0.05 versus the sham group; ^#^*p < *0.05 versus the RM and RM + BM group (n = 6). (**b**) Periodic acid-Schiff-stained sections of mouse kidneys (200×). Sham: Normal kidney section. RM(rhabdomyolysis): Kidney section of glycerol-treated mouse showing tubular necrosis and cast formation. RM + BM: Kidney section of glycerol-treated mouse and biological membrane(BM)-wrapped kidney also showing tubular necrosis and cast formation. RM + BM + MSCs: Kidney section of biological membrane-packed mesenchymal stem cells(MSCs) on the renal tissue and glycerol-treated mouse showing morphological damage significantly improved. Note: ^*^p < 0.05 versus the RM group; ^†^*p < *0.05 versus the RM + BM group; ^#^*p < *0.05 versus the sham group (n = 8). (**c**) Images of the transplanting MSCs-GFP under biological membrane. MSCs-GFP survived on the surface of renal parenchyma. GFP-labelled MSCs (green) were detected using two-photon fluorescence confocal microscopy.

Glycerol administration resulted in significant increases in serum creatinine(Scr) and blood urea nitrogen(BUN) in the RM mice compared with the levels in the sham group. We used 10^6^ biological membrane-packed MSCs on the renal tissue. This method markedly reduced serum creatinine (SCr) and blood urea nitrogen (BUN) (Fig. 1a) 24, 48 and 72 hours after glycerol injection compared with the levels in the RM and RM + BM groups. We selected the 72-hour time point(the most severest AKI) to evaluate kidney injury. Sham mice did not show any significant tubular damage. RM and RM + BM mice showed tubular necrosis, tubular dilatation and cast formation. RM + BM + MSCs mice demonstrated markedly improved tubular injury (Fig. 1b).

To evaluate survival of transplanted MSCs, we studied additional animals after transplanting MSCs-GFP in RM + BM + MSCs-GFP mice group. We found MSC-GFP survived on the surface of renal parenchyma but without migrating to parenchyma of kidneys (Fig. 1c).

### Biological membrane-packed MSCs increase the expression of E-cadherin in renal tubular epithelial cells of glycerol-inducedmouse kidney injury

**Figure 2:**
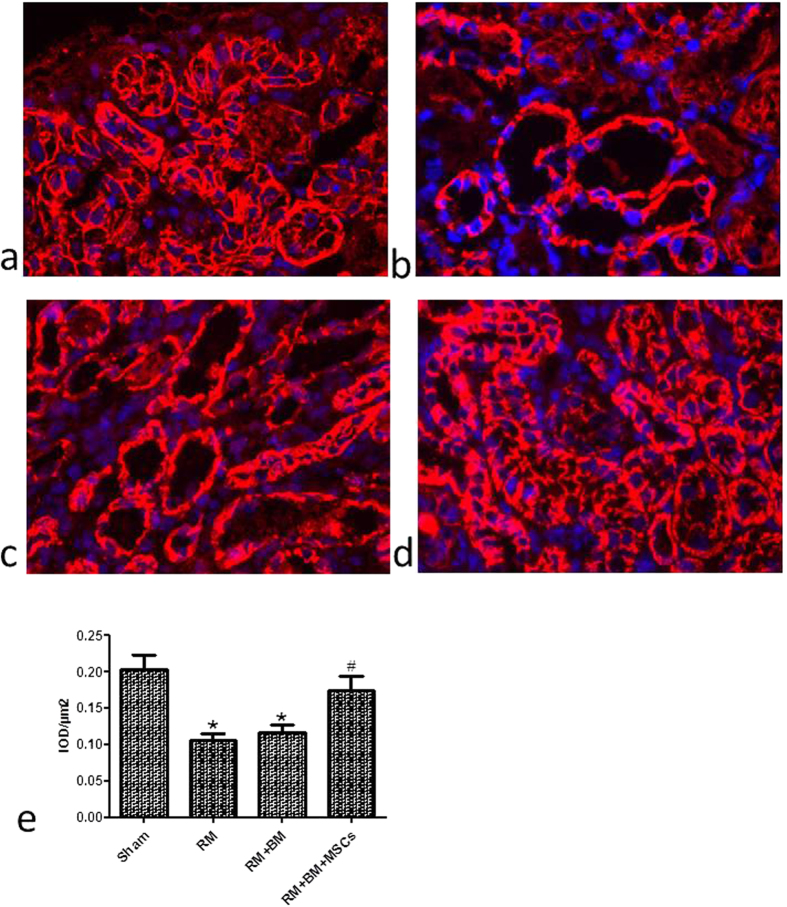
Expression of E-cadherin in kidney frozen sections and tissues. The localization of E-cadherin in frozen sections was determined by immunofluorescencestaining (IF) (400×) with E-cadherin antibody (red) in sham group (**a**); blue represents the nucleus. The expression of E-cadherin was distributed abnormally and reduced in renal tissue of RM(rhabdomyolysis) (**b**) and RM + BM(biological membrane) mice (**c**). Biological membrane-packed mesenchymal stem cells on the renal tissue appear to be better preserved (**d**). (**e**) Quantitative analysis of staining intensity showed a higher expression of E-cadherin in RM + BM + MSCs group compared with RM and RM + BM groups. Note: *p < 0.05 versus the sham group, ^#^p < 0.05 versus the RM group, n = 6.

E-cadherin is a cell adhesion molecule that plays an important role in maintaining renal epithelial polarity and integrity. E-cadherin is expressed in all tubular segments of mouse kidney. E-cadherin was expressed in most of the tubular epithelial cells of the mice in the sham group (Fig. 2a), while it was significantly decreased in the RM and RM + BM groups (Fig. 2b,c). Biological membrane-packedmesenchymal stem cells on the renal tissueappeared to be better preserved than in the RM and RM + BM mice groups (Fig. 2d) by immunofluorescence staining. Quantitative analysis of staining intensity showed a higher expression of E-cadherin in RM + BM + MSCs group compared with RM and RM + BM groups (Fig. 2e).

### Biological membrane-packed MSCs relieve glycerol-induced tubular apoptosis in mice

**Figure 3:**
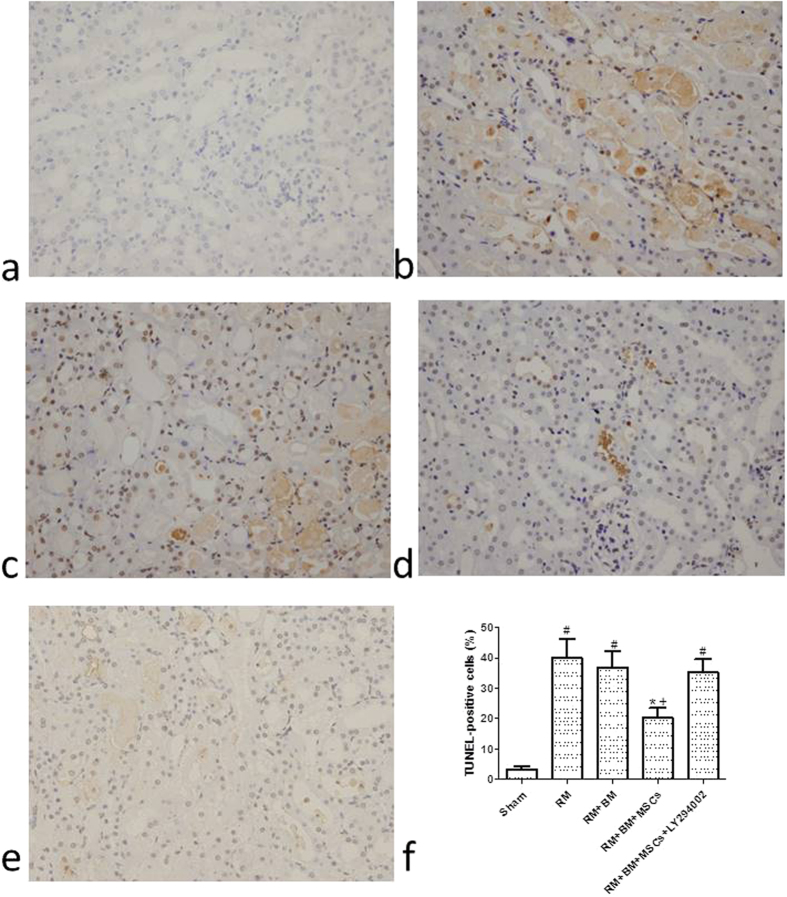
Determination of tubular cell apoptosis *in situ* terminal deoxynucleotidyl transferase biotin-dUTP nick end-labeling (TUNEL) assay (200×). (**a**) Few apoptotic cells were observed in the sham group. The RM group (**b**) and the RM + BM group (**c**) displayed markedly more TUNEL-positive cells, mainly in the corticomedullary junction and medullary area. (**d**) Biological membrane-packed mesenchymal stem cells on the renal tissue treatment decreased the number of TUNEL-positive cells, and fewer apoptotic cells were observed. the RM + BM + MSCs + LY294002 group (**e**) also displayed TUNEL-positive cells. (**f**) The indices of apoptosis in the different groups were consistent with the TUNEL staining results. Note: ^*^p < 0.05 versus the RM group; ^†^*p *< 0.05 versus the RM + BM group; ^#^*p *< 0.05 versus the sham group (n = 8).

In the TUNEL assay, the nuclei of TUNEL-positive cells were stained brown, indicating apoptotic cells. Few apoptotic cells were observed in the sham group (3.31 ± 0.091) (Fig. 3a), whereas the RM group displayed markedly more TUNEL-positive cells than the sham group (39.96 ± 6.29) (Fig. 3b); the RM + BM group also displayed TUNEL-positive cells (36.86 ± 5.29) (Fig. 3c), which were observed mainly in the corticomedullary junction and medullary area, with occasional occurrence in the cortex. Some TUNEL-positive cells were detached from the tubular basement membrane in the lumen. Biological membrane-packed MSCs on the renal tissue treatment decreased the number of TUNEL-positive cells, and fewer apoptotic cells were observed in the RM + BM + MSCs group (Fig. 3d) compared with the RM group and RM + BM group (20.25 ± 3.21 vs. 39.96 ± 6.29 and 20.25 ± 3.21 vs. 36.86 ± 5.29., respectively, *p *< 0.05).

### Function of mitochondria

**Figure 4:**
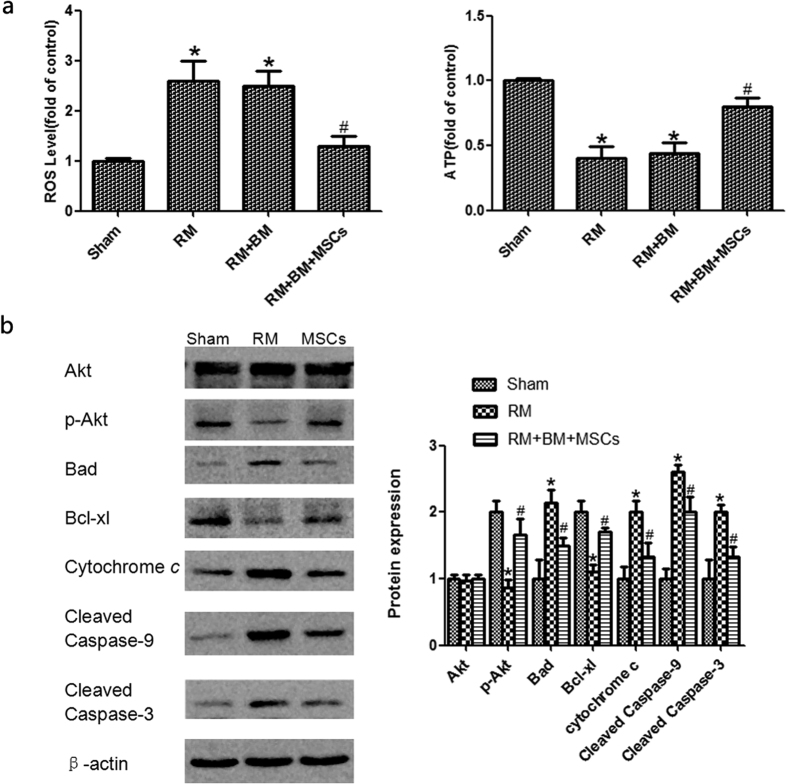
Mitochondrial function changes and p-Akt, Akt, Bad, Bcl-xl, Cytochrome c, cleaved Caspase-9 and cleaved caspase-3protein expression. (**a**) Reactive oxygen species (ROS) relative level in renal tubules; and ATP relative level in renal tubular mitochondria. (**b**) The expression of total Akt protein in each group was at the same level, while p-Akt was significantly lower in the RM group compared with that of the sham group, and p-Akt was significantly upregulated in the RM + BM + MSCs group compared with that of the RM group. Bad, Cytochrome c, cleaved caspase-9, and cleaved caspase-3 were significantly higher in the RM group than in the sham group. MSC treatment significantly reduced Bad, Cytochrome c, cleaved caspase-9, and cleaved caspase-3 compared with the RM group. Bcl-xl was significantly lower in the RM group compared with the sham group, and Bcl-xlwas significantly upregulated in the RM + BM + MSCs group compared with the RM group. Note: *p < 0.05 versus the sham group, ^#^p < 0.05 versus the RM group, n = 6.

RM increased the production of ROS. In the RM + BM + MSCs group, ROS production significantly decreased. This result is indicative of the protective effect of MSCs on mitochondrial functions. About 72 hours after model induction, we also found that ATP, the marker of respiratory function in the mitochondria, markedly decreased, indicating that RM suppresses mitochondrial respiratory function. At both time points, ATP production in the RM + BM + MSCs group treated group was higher than that in the RM group, revealing the beneficial effects of MSCs in alleviating RM induced impairment of mitochondrial respiratory function (Fig. 4a).

### Biological membrane-packed MSCs influence the amountsof Akt, p-Akt, and apoptosis-related proteins in mice with glycerol-induced kidney injury

The PI3K/Akt pathway is an important signaling pathway for cell survival. To research the function of the PI3K/Akt pathway in rhabdomyolysis-induced AKI, we assessed the Akt and p-Akt protein expression in the sham, RM and RM + BM + MSCs groups (Supplementary Fig. S1). The expression of totalAkt protein in each group was at the same level, while p-Akt was significantly lowerin the RM group compared with the sham group and was significantly upregulated in the RM + BM + MSCs group compared with the RM group. Activated Akt (p-Akt) further phosphorylates downstream signaling molecules, such as Bad, which results in the inhibition of their activities. Bad, as a member of the Bcl-2 family, associates with Bcl-xl in the mitochondria, influencing the caspases. The pro-apoptotic Badand the anti-apoptotic Bcl-xl may play some role in rhabdomyolysis-induced AKI. Caspase-3 acts as an “executioner” proteinin the apoptotic pathway. Caspases-9 are referred to as “initiator caspases” as they are closely coupled to upstream, pro-apoptotic signals^14^. Cleaved caspase-3 and -9 represent the activated status of these proteins, which are involved in a mitochondrial-dependent pathway that eventually leads to apoptosis. The downregulation of Bad andcaspase-3 and -9 and the upregulation of Bcl-xl were observed in the RM + BM + MSCs group comparedwith the RM group (Fig. 4b).

### MSCs attenuate myoglobin-induced apoptosis in HK-2 cells by activating the PI3K/Akt pathway and inhibiting apoptosis pathway

**Figure 5:**
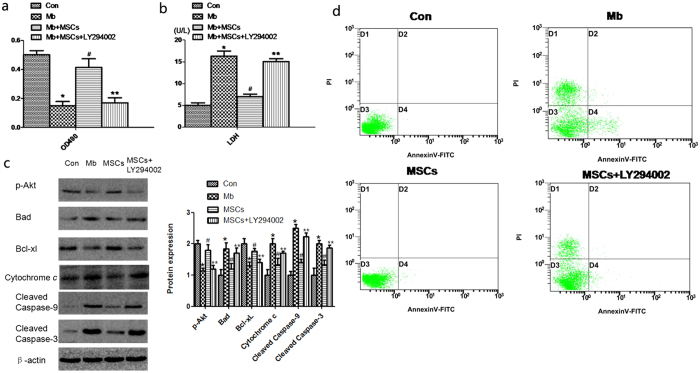
Effect of MSCs and LY294002on the cell viability, cell injury, protein expressions and Annexin V-FTIC/PI double-staining flow cytometry of myoglobin-induced apoptosis in HK-2 cells. (**a**) The MTT assay showed the cell viability of HK-2 cells. (**b**) Supernatant LDH release tests indicated cell injury of HK-2. (**c**) p-Akt, Bad, Bcl-xl, Cytochrome c, cleaved caspase-9 and cleaved caspase-3 were measured by western blot. (**d**) Cell apoptosis was evaluated by AnnexinV-FTIC/PI double-staining flow cytometry. Note: *p < 0.05 versus the Con group, ^#^p < 0.05 versus the Mb group, **p < 0.05 versus the MSC group n = 6.

We next investigated myoglobin-induced apoptosis in HK-2 cells *in vitro* to discover the mechanisms of the PI3K/Akt pathway and apoptosis pathway involved in rhabdomyolysis-relatedAKI. The HK-2 cells were incubated in normal media (Con), 200 mM ferrous myoglobin media (Mb), and MSCs plus ferrous myoglobin (Mb + MSCs), MSCs added LY294002 plus ferrous myoglobin (Mb + MSCs + LY294002). The MTT assay showed that the cell viability of HK-2 incubated with ferrous myoglobin was lower than that of the control (0.16 ± 0.06 vs. 0.50 ± 0.04). LY294002 reversed the increased viability of HK-2 cells mediated by MSC treatment (0.17 ± 0.05 vs. 0.41 ± 0.09) (Fig. 5a). Supernatant LDH release tests indicated that the LDH release of HK-2 incubated with ferrous myoglobin was higher than that of the control (17.29 ± 1.05 vs. 5.01 ± 0.46 U/L). Supernatant LDH release of HK-2 in the Mb + MSCs + LY294002 group was higher than in the Mb + MSC group (15.03 ± 0.67 vs. 7.03 ± 0.44 U/L) (Fig. 5b).

In another experiment, HK-2 cells were incubated for 24 h in the Con, Mb, Mb + MSC and Mb + MSC + LY294002 groups. The total protein of the cells was extracted, and apoptosis-related protein expression was measured by western blot (Supplementary Fig. S2). We found that myoglobin incubation increased Bad, Cytochrome c, cleaved caspase-9, and cleaved caspase-3 and decreased p-Akt and Bcl-xl, while the MSCs plus myoglobin further decreased Bad, Cytochrome c, cleaved caspase-9, and cleaved caspase-3 and increased p-Akt and Bcl-xl compared to ferrous myoglobin media. The addition of LY294002 inhibited the effects of MSC treatment (Fig. 5c).

Apoptosis was also evaluated by flow cytometry. HK-2 cells were incubated for 24 h with different groups. AnnexinV-FTIC/PI double-staining flow cytometry showed that ferrous myoglobin induced apoptosis, while the incubation with MSCs decreased cell apoptosis. Added LY294002 increased cell apoptosis (Fig. 5d); the apoptosis rates are listed in Table 1.

### LY294002 reverses the effects of MSCs on renal mitochondria-related apoptosis after glycerol-induced AKI in a mouse model

**Figure 6:**
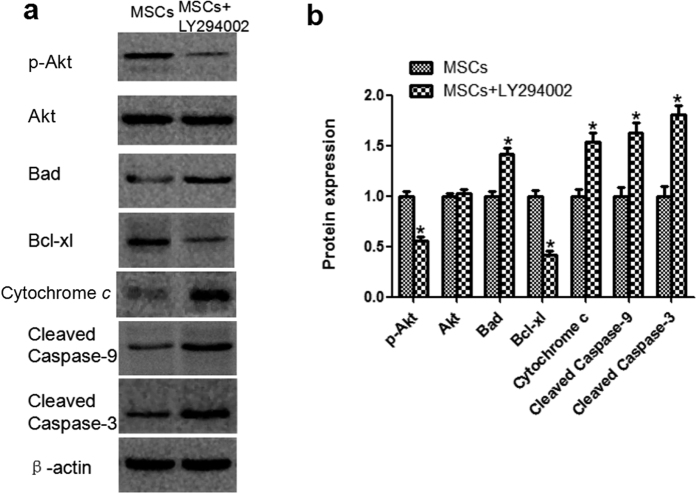
Effect of LY294002 on Akt phosphorylation and renal expression of mitochondria apoptotic proteinsin the kidneys of glycerol-induced AKI with MSCs treatment. (**a**) The results of western blotting indicated that LY294002 significantly reduced MSC-induced phosphorylation of p-Akt. (**b**) The analytic outcomes show that LY294002 pretreatment significantly increased Bad, Cytochrome c, cleaved caspase-9, and cleaved caspase-3 and decreased Bcl-xl compared with those of the MSC group. Note: *p < 0.05, n = 6.

To confirm that MSCs attenuate glycerol-induced AKI though the PI3K/Akt and apoptosis pathways, LY294002 was injected intraperitoneally to a glycerol-induced AKI mousemodel *in vivo*. the RM + BM + MSCs + LY294002 group also displayed TUNEL-positive cells (34.79 ± 4.82) (Fig. 3e). LY294002 decreased the expression ratio of p-Akt, based on the results of the western blotting. Moreover, LY294002 reversed the expression changes in mitochondrial-apoptosis related proteins (Bcl-xl, Cytochrome c, cleaved caspase-9, -3) mediated by MSCs treatment (Fig. 6 and Supplementary Fig. S3)). These changes indicated that the protective effect of MSCs on glycerol-induced AKI could be abolished by the inhibitory effects of LY294002 on the PI3K/Akt and mitochondrial apoptosis signaling pathways.

## Discussion

Rhabdomyolysis causes high mortality and is accompanied by a series of clinical and laboratory abnormalities resulting from the lysis of striated muscle cells^15^. While technological advances enable treatments for many diseases, we continue to encounter traumatic rhabdomyolysis as a result of wars, terror attacks, earthquakes, and other natural disasters^16^,^17^. Mesenchymal stem cells are one of the few stem cell types that have been applied as therapeutic agents for immunomodulation and tissue repair, which highlights their potential for recovery from kidney injury^6^. The ability of MSCs to accomplish repair via paracrine and/or endocrine mechanisms, such as inhibiting the release of pro-inflammatory cytokines and secreting a variety of trophic growth factors^18^, may collectively mediate the protective and regenerative effects of AKI in laboratory rodents^19^. Duragen is a collagen matrix synthesized from bovine Achilles tendon. It is a type of biological membrane that can be used to treat cerebrospinal fluid leaks^13^. We used Duragen as a biological membrane to wrap the MSCs on the glycerol-induced kidney to prevent MSC leakage and to allow the MSCs to survive in the body for longer periods of time.

This study showed that biological membrane packing of MSCs on the kidney improved renal function in rhabdomyolysis-induced AKI. By 72 hours, the glycerol-treated rats developed AKI and exhibited significantly increased Scr and BUN levels and significant morphological changes, including vacuolar degeneration, brush border loss, cast formation, severe tubular necrosis and tubulorrhexis of many proximal tubules. In contrast, with the biological membrane-packed MSCs on the renal tissue, the Scr and BUN levels, renal tubular epithelial cell degeneration and necrosis, and tubule necrosis scores were all significantly reduced. However, there was no significant difference between the glycerol-induced AKI group and that with only biological membrane packing on the renal tissue. Therefore, the biological membrane played a minimal role between the glycerol-induced AKI group and the biological membrane packing on the renal tissue group. Renal tubular epithelial cell apoptosis is important in the pathogenesis of rhabdomyolysis-induced AKI. In our study, TUNEL staining revealed numerous apoptotic cells in the kidneys of glycerol-induced AKI rats, whereas the number of apoptotic cells was significantly decreased in the mice treated with biological membrane-packed MSCs on the renal tissue, suggesting that treatment may significantly reduce apoptosis. Western blot results showed that the downregulation of Bad, cytochrome *c*, caspase-9, and caspase-3 and the upregulation of Bcl-xl and p-Akt were observed in the MSC group compared with the RM group. This result showed that MSC treatment could significantly reduce apoptosis.

In AKI caused by different etiologies, the reduction in the number of tubular epithelial cells due to excessive apoptosis or necrosis is a major intrinsic change; impairment of the mitochondria has been identified as the primary cause of excessive apoptosis and necrosis^20^. After the mitochondria are damaged, the respiratory complex breaks down, the membrane potential is altered and permeability increases, followed by the release of proapoptotic factors, such as cytochrome *c*.^21^ Nath *et al*. confirmed that renal mitochondrial respiration is disrupted and further diminishes after glycerol injection^22^. We found that 72 hours after glycerol injection, energy production in the mitochondria decreased, accompanied by an increase in the production of ROS and the release of proapoptotic factors; these changes led to the increased apoptosis of tubular epithelial cells. These changes indicated that cell apoptosis following the dysfunction of the mitochondria in RM induced the development and progression of AKI. Our results also showed that ATP production in the RM + BM + MSCs group treated group was higher than that in the RM group, revealing the beneficial effects of MSCs in alleviating RM induced impairment of mitochondrial respiratory function. At the same time, we suspect that the mitochondrial (intrinsic) apoptotic pathway may play a significant role in the process of AKI and MSCs may reduce rhabdomyolysis-associated renal injury through the mitochondrial apoptosis pathway.

The present study suggested that packing MSCs on the renal tissue improved kidney function and ameliorated the renal injury induced by glycerol. Our study also focused on the anti-apoptotic mechanisms responsible for the therapeutic effects of MSCs in glycerol nephropathy. The PI3K/Akt signaling pathway regulates cell survival. Akt is an essential kinase downstream of PI3K. Phosphorylation of Akt is necessary for its activation, which subsequently regulates many cellular responses. Akt also plays a crucial role in suppressing apoptosis. As a survival factor, activated Akt (p-Akt) further influences downstream signaling molecules, such as Bad, which results in the inhibition of their activities and further reduces the expression of pro-apoptotic proteins^23^. Activated Akt inhibits pro-apoptotic Bad and Bax levels and elevates Bcl-2 and Bcl-xl expression^24^. Bad and Bcl-xl are members of the Bcl-2 family. In the intrinsic or mitochondrial pathways, the Bcl-2 family plays a crucial role in the control of apoptosis and can be classified into two functionally distinct groups: pro-apoptotic proteins, such as Bax, Bad, and Bid, and anti-apoptotic proteins, such as Bcl-2 and Bcl-xl. Their interplay tightly regulates the mitochondrial apoptosis pathway^25^. Bad associates with Bcl-xl in the mitochondria to form a pro-apoptotic complex to further influence the release and activation of downstream pro-apoptotic proteins, such as caspases^26^. Cleaved caspase-9 and cleaved caspase-3 are the activated versions of these proteins that are involved in a mitochondrial-dependent pathway that eventually leads to apoptosis. Caspase-9 cleaves caspase-3 to initiate apoptosis^25^.

To elucidate the regulatory details of MSC application, we investigated the apoptosis of renal cells induced by glycerol both *in vitro* and *in vivo. In vitro*, we induced apoptosis in HK-2 cells through the addition of ferrous myoglobinto replicate rhabdomyolysis-induced AKI. Treatment with myoglobin led to a decrease in p-Akt and the degradation of anti-apoptotic Bcl-xl, whereas pro-apoptotic Bad, cleaved caspase-9, and cleaved caspase-3 were increased. Administration of MSCs upregulated p-Akt and Bcl-xl and downregulated Bad, cleaved caspase-9, and cleaved caspase-3, which corresponded to reduced apoptosis. With the administration of the PI3K inhibitorLY294002, the protective effects of MSCs were reversed. Combined with the MTT assay, LDH and AnnexinV-FTIC/PI double-staining flow cytometry results suggest that MSCs attenuate myoglobin-induced apoptosis in HK-2 by activating the PI3K/Akt pathway and inhibitingthe mitochondrial pathway of apoptosis. In the *in vivo* study, we also used a glycerol-induced mouse AKI modelto understand the mechanisms responsible for the renoprotective effects of MSCs. The expression of total Akt protein in each mouse group was similar, while p-Akt was decreased in the RM group and significantly upregulated in the MSC-treated group. In contrast, p-Akt was decreased when LY294002 was added. This showed that MSCs could regulate the PI3K/Akt pathway. MSCs increased p-Akt, which resulted in further decreases in Bad, cleaved caspase-9, and cleaved caspase-3 and increases in Bcl-xl. However, LY294002 pretreatment largely reversed the protective effects of MSCs, as well as the expression of signals in the mitochondrial-dependent pathway. Based on the present results, we presume that MSCs attenuate glycerol-induced AKI by regulating the PI3K/Akt and mitochondrial apoptosis pathways. MSCs could reduce rhabdomyolysis-associated renal injury and cell apoptosis by activating the PI3K/Akt and inhibitingthe mitochondrial apoptosis pathways.

Biological membrane packing of MSCs on the renal tissue slowing the progression of AKI is a new method for mesenchymal stem cells treating AKI. This method have distinguishing characteristic compared to other methods. Cheng *et al*. transplanted MSCs via renal subcapsular route. They showed that subcapsular transplantation of MSCs provided protection against cisplatinum-induced AKI. But one problem had to be indicated is that this method may cause renal subcapsular hematoma. Renal subcapsular hematoma is a common clinical disease. Since the space between renal fascia and parenchyma is narrow, a small amount of bleeding will have a great oppression to renal tissue. A large amount of bleeding for a long time on the renal parenchymal is more likely to damage the remaining renal function and cause hypertension or infection. Our results demonstrated that biological membrane packing of MSCs on the renal tissue had a therapeutic function on glycerol-induced AKI. The difference between biological membrane and subcapsular route needs to be further studied. But our method provides a broader space for cells location and avoids the renal injury caused by bleeding and pressure^27^. Geng *et al*. demonstrated that intravenous infusion of MSCs after induction of rhabdomyolysis ameliorated renal function impairment and severe tubular injury^28^. Despite significant improvement in renal histology and function, most cells were located in the lungs or injured muscle, and none were present in the kidney. Even following direct administration into the kidney via the renal artery, MSCs were only transiently located within the glomerular capillaries or interstitium. The biological membrane-assisted cell therapy system holds great promise for achieving cell location and migration at the lesion site as a widely adopted platform technology. Both intravenous infusion of MSCs and biological membrane packing of MSCs on the renal tissue have a therapeutic rescue function. The difference can be futher studied. On the basis of our findings, we also believe that the biological membrane could help to preserve the kidney and also offer a widely applicable cell delivery platform technology to boost the healing power of cell therapy.

In conclusion, the present study offers a new perspective in stem cell therapy in which mesenchymal stem cells could play a beneficial therapeutic role. At the same time, we demonstrated that the infusion of MSCs protects against glycerol-induced AKI by inhibiting cell apoptosis. The beneficial effects are due to its abilities to activate the PI3K/Akt pathway and inhibit apoptosis by modulating mitochondrial-apoptotic pathways. Our study provides a preliminary understanding of the role of MSCs in the treatment of AKI.

## Methods

This study was designed and carried out in accordance with the Guide for the Care and Use of Laboratory Animals of the Chinese PLA General Hospital and Military Medical College. The research protocols were approved by the Animal Ethics Committee of the Chinese PLA General Hospital and Military Medical College. All methods were carried out in accordance with the approved guideline.

### Experimental animals and procedures

Eight- to twelve-week-old C57BL/6 male mice were provided by the Experimental Animal Center of the Academy of Military Medical Sciences. The mice were housed at a stable temperature in a 12-hour light/dark cycle. We provided adequate standard rodent chow and water. The animals were acclimated for seven days before initiating the experiment. All animal protocols were approved by the Animal Ethics Committee of the Chinese PLA General Hospital and Military Medical College. C57BL/6 mice were deprived of water for 24 hours and then administered half the dose of glycerol (50% v/v in sterile saline) in each hindlimb muscle following mild sedation with pentobarbital. Our dose-dependent studies defined the optimal dose of glycerol as 8 mL/kg body weight. Six hours later, the mice received kidney capsule peeled off and biological membrane packing the renal tissue or biological membrane-packed mesenchymal stem cells (MSCs) on the renal tissue. Animals used in this study were cared for in accordance with the guidelines of the Chinese PLA General Hospital and Military Medical College.

The following groups were evaluated: sham, control plus peeling off the kidney capsule; RM, rhabdomyolysis(RM) with glycerol administration plus peeling off the kidney capsule; RM + BM, rhabdomyolysis plusDuragen as a BM(Integra Life Sciences, Plainsboro, NJ, USA); rhabdomyolysis plus peeling off the kidney capsule and biological membrane packing the renal tissue; RM + BM + MSCs, rhabdomyolysis plus peeling off the kidney capsule and 10^6^ biological membrane-packed mesenchymal stem cells (MSCs) on the renal tissue. Each experimental group comprised 8 mice. At various time points after rhabdomyolysis, blood and kidney samples were harvested for further processing.

The pharmacological inhibitor of PI3K, LY294002, has been used to analyze the role of the PI3K/Akt pathway in rhabdomyolysis-induced AKI. We added the RM + BM + MSCs + LY294002 group: rhabdomyolysis plus peeling off the kidney capsule and 10^6^ biological membrane-packedMSCs on the renal tissue plus LY294002 (40 mg/kg) (Sigma, St. Louis, MO, USA) injected intraperitoneally in a total volume of 500 μl in a solution containing 10% (v/v) DMSO after the operation completed. DMSO was used in all mice as a vehicle control.

### Cell loading into Duragen and Operation Procedure

The biological membrane of Duragen was purchased from Integra Lifesciences Company. The Duragen was sterile. MSCs were isolated and cultured in the MSC growth medium. 10^6^ MSCs suspension were subsequently pipetted onto 2.5 × 1.5 cm^2^ Duragen and automatically absorbed to the material. Then, the kidney capsule was peeled off. We wrapped up the the renal tissue by Duragen which contained MSCs. We stitched the edge of the Duragen and kept a gap for renal pedicle. After that, the kidney was put back into the abdominal cavity. Both of the kidneys were conducted by this procedure.

### Ethical approval

All animal protocols were approved by the Animal Ethics Committee of the Chinese PLA General Hospital and Military Medical College. Animals used in this study were cared for in accordance with the guidelines of the Chinese PLA General Hospital and Military Medical College.

### MSC cell culture

C57BL/6 mouse bone marrow–derived MSCs and GFP-labeled MSCs derived from the bone marrow of C57BL/6 mice were obtained from Cyagen Biosciences (Cyagen Biosciences, Sunnyvale, CA, USA) and used according to the manufacturer’s instructions. Identification of the cells according to the cell surface phenotypes was performed by the supplier. The cell surface phenotypes were CD44 + , CD29 + , SCA-1 + and CD117-. The certificate of analysis for MSCs and GFP-labeled MSCs can be seen in Supplemental Material. MSCs were placed in 25-cm^2^ culture flasks and cultured with MSC growth medium (Cyagen Biosciences) at 37 °C under 5% CO_2_ and 90% humidity. The medium was changed every two days. The sixth- to eighth-passage MSCs were used for experiments.

### Identification of transplanted MSCs

The viability and fluorescence of the transplanted MSCs-GFP were assessed and imaged using a Leica two-photon fluorescence confocal imaging TCS SP5 system (Leica Microsystems, Mannheim, Germany).

### Histopathological examination for acute tubular necrosis scores

The kidneys were fixed in 10% formalin for 24 hours and underwent routine dehydration and paraffin embedding. Renal tissues were sectioned at 3-μm thickness and stained with periodic acid–Schiff (PAS) using standard methods. Histological examinations were performed in a blinded fashion.

The severity of acute tubular necrosis was quantified by counting the percentage of tubules that displayed cell necrosis, loss of brush border, cast formation and tubule dilatation as follows: 0 = none; 1 = ≤10%; 2 = 11% to 25%; 3 = 26% to 45%; 4 = 46% to 75%; and 5 ≥ 76%^29^. Approximately 80 high-power fields (HPFs, × 200) per individual mouse (20 HPFs per slide, four slides per animal) were evaluated (n = 8 per group).

### Tissue immunofluorescence staining

Frozen sections of 4-μm thickness were prepared and washed with PBS twice for 10 min and pre-incubated in 10% casein (Vector, Burlingame, CA, USA) in PBS for 30 min. The sections were incubated in E-cadherin antibody (1/50, Abcam, Cambridge, MA, USA) overnight in a moisture chamber and then washed sufficiently with phosphate-buffered saline-Tween (PBST) to remove unbound antibody. Next, the sections were incubated with Cy3-conjugated anti-rabbit IgG antibody (Jackson ImmunoResearch, Baltimore, PA, USA) for 60 min at room temperature and washed as described for the primary antibody. Finally, the preparations were washed with PBS and mounted with fluorescent mounting medium containing 4′,6-diamidino-2-phenylindole (DAPI) (Zhongshan Goldenbridge Biotechnology, Beijing, China). The sections were mounted on glass slides and analyzed under an Olympus EX71 fluorescence microscope equipped with an Olympus DP72 digital camera (Olympus, Tokyo, Japan) at a magnification of 400x. Renal sections were randomly assessed and photographed and coded under the fluorescence microscope. The fluorescent microscope images were assessed using Image-Pro Plus 6.0 software, and the integrated optical density (IOD) of each photograph was collected. 6 fields of each slice were randomly selected for blinded measuring (6 slices in each group). Images were quantified by the immunoreactive area (IA) in μm^2^ and the integrated optical density (IOD). Staining intensity (SI) for each image was calculated as SI = IOD/IA and mean with standard deviation was obtained for each series.

### *In situ* TUNEL assay

We performed a terminal deoxynucleotidyl transferase-mediated deoxyuridine triphosphate nick end labeling(TUNEL) assay according to the manufacturer’s instructions (Merck Millipore, Billerica, MA, USA). Kidney tissue was fixed in 10% formalin overnight, dehydrated, embedded in paraffin, cut at 2-mm-thick sections, and placed on a numbered polylysine-coated glass slide. TUNEL-positive cells, which were stained brown, were counted under 200x magnification. Nuclei were stained with hematoxylin to observe the nature of the TUNEL-positive cells. Six to eight fields per section and two to three sections per kidney were examined in each experiment. We calculated the percentage of TUNEL-positive cells relative to the total number of renal tubular cells as the apoptotic rate.

### Determination of ROS production in kidney tissue

Mouse kidney homogenate was analyzed fluorometrically by measuring the oxidation of the nonfluorescent probe 20, 70-dichloro-fluorescein diacetate (DCFDA) into the fluorescent metabolite DCF as described by Topo *et al*.^30^. In brief, 30 mL of kidney homogenate in PBS was mixed with 5 μm DCFDA and incubated for 30 minutes at room temperature. The mean fluorescence intensity was directly measured at excitation and emission wavelengths of 485 and 535 nm, respectively.

### ATP measurement

Renal cortical tissue ATP was measured as described by Vives-Bauza *et al*.^31^. ATP was extracted from flash frozen kidney cortex with 0.4 M HClO4. ATP levels were determined using an ATP bioluminescence assay kit (Roche) and normalized to protein concentration.

### Western analysis

Rabbit polyclonal anti-Bcl-xl, rabbit polyclonal anti-Bad, rabbit polyclonal anti-cytochrome *c.* rabbit polyclonal anti-cleaved caspase-9, rabbit polyclonal anti-cleaved caspase-3, rabbit polyclonal anti-Akt and rabbit polyclonal anti-p-Akt, horseradish peroxidase labeled goat anti-rabbit immunoglobulin G (IgG), and rabbit anti-mouse IgG were purchased from Abcam (Abcam, Cambridge, MA, USA). Mouse anti-β-actin monoclonal antibody was obtained from Sigma (St. Louis, MO, USA). Frozen kidney tissue was homogenized for radioimmunoprecipitation. The homogenates were clarified by centrifugation at 12,000 g for 10 min at 4 °C, and the supernatants were collected. Supernatant samples (70 μg) were subjected to 15% polyacrylamide gel electrophoresis and transferred to cellulose acetate membranes. The membranes were blocked with 1 × casein solution for approximately 4 hours and then incubated with rabbit polyclonal anti-cleaved caspase-3 and anti-cleaved caspase-9, anti-Bcl-xl and anti-Bad, or anti-Akt and p-Akt antibodies overnight at 4 °C. The blots were washed with Tris-buffered saline-Tween-20 (TBST) buffer and subsequently incubated with goat anti-rabbit or rabbit anti-mouse IgG. After washing with TBST, the blots were developed with enhanced chemiluminescence reagents. A mouse monoclonal anti-β-actin antibody was used as a control for each sample. Signals of the blots were analyzed by the free image analyzing software Image J 1.42 (downloaded from http://rsbweb.nih.gov/ij/download.html).

### HK-2 cells culture and ferrous myoglobin media

Myoglobin (M0630, Sigma Aldrich Corporation, St. Louis, MO, USA) and ascorbic acid (Sigma) were dissolved in Dulbecco’s Modified Eagle Medium(DMEM):Nutrient Mixture F-12 media(DMEM/F12, Gibco, Life Technologies Corporation, Carlsbad, CA, USA) to make the storage solution. The storage solution was diluted to a proper concentration just before each experiment. A concentration of 200 mM (i.e., 3.6 g/L) ferrous myoglobin was used in this study. Only reduced myoglobin is cytotoxic, but only metmyoglobin was commercially available, so the myoglobin and ascorbic acid solutions were mixed to make the ferrous myoglobin mediumas described in the literature^32^. The final concentration of myoglobin was 200 mM, while ascorbic acid was 2 mM. The ascorbic acid had reduced myoglobin to ferrous status when the color changed from brown to reddish.

The following groups were evaluated: Con, HK-2 cells in normal media; Mb, HK-2 cells in ferrous myoglobin medium; Mb + MSCs, HK-2 cells in ferrous myoglobin medium co-culture with MSCs in 6- or 96-well transwell plates. Mb + MSCs + LY294002, HK-2 cells in ferrous myoglobin medium were co-culture with MSCs in 6- or 96-well transwell plates with the PI3K inhibitor, LY294002 (25 μmol/L) (Sigma).

### Cell viability and injury

The HK-2 cells were cultured in 96-well transwell plates. After synchronization for 16 h with low-serum medium, cells in the Con, Mb, Mb + MSC and Mb + MSC + LY294002 groups were incubated for 24 hours. The cells were then washed and determined by 3-(4,5)-dimethylthiahiazo(-z-y1)-3,5-diphenyltetrazolium bromide (MTT) assay. The culture medium was transferred carefully to another test tube and centrifuged (1200 rpm, 5 min), after which supernatant lactose dehydrogenase (LDH) was measured.

### Cell apoptosis flow cytometry evaluation

The HK-2 cells were cultured in 6-well transwell plates. After synchronization, the cells in the Con, Mb, Mb + MSC and Mb + MSC + LY294002 groups were incubated for 24 hours. Collected cells were double-stained with AnnexinV-FITC and PI (BD Biosciences, Franklin Lakes, NJ, USA), for the flow cytometry test of early apoptosis(Annexin V-positive).

### Cell protein expression

The HK-2 cells were cultured in 6-well transwell plates. After synchronization, cells were allocated to the Con, Mb, Mb + MSC and Mb + MSC + LY294002 groups. At 24 hours of incubation, the cellular protein was extracted. Then, p-Akt, p-Bad, Bcl-xl, cytochrome *c,* cleavedcaspase-9, cleaved caspase-3, and β-actin protein expression were determined bywestern blot assay. Protein expression was measured by examining the relative intensity of bands. All the antibodies were purchased from Abcam (Abcam, Cambridge, MA, USA).

### Statistical analysis

Statistical analysis was performed using IBM SPSS Statistics 17.0.2 software (IBM Corporation, Armonk, NY, USA). Data were expressed as mean values ± standard deviation (SD) for quantitative parametric measures in addition to median percentiles for quantitative non-parametric measures. All data were tested for normal distribution by Shapiro-Wilk test. Multiple comparisons of parametric data were performed using one-way analysis of variance (ANOVA) followed by Student–Newman–Keuls post-hoc tests. Student’s t-test was used to compare differences between means. In the cases where the requirements of parametric analysis were not satisfied, the non-parametric Wilcoxon test was used. Wilcoxon Rank Sum test was used for comparison between two independent groups for non-parametric data. Wilcoxon signed rank test was used for comparison between two dependent groups for non-parametric data. A P-value of <0.05 was considered significant.

## Additional Information

**How to cite this article**: Geng, X. *et al*. Biological Membrane-Packed Mesenchymal Stem Cells Treat Acute Kidney Disease by Ameliorating Mitochondrial-Related Apoptosis. *Sci. Rep.* **7**, 41136; doi: 10.1038/srep41136 (2017).

**Publisher's note:** Springer Nature remains neutral with regard to jurisdictional claims in published maps and institutional affiliations.

## Supplementary Material

Supplementary Materials

## Figures and Tables

**Table 1:** Cell apoptosis of each group.

Items	Apoptosis rate (%)
Con	0.15 ± 0.06
Mb	10.68 ± 2.24^*^
MSCs	0.98 ± 0.09^#^
MSCs + LY294002	8.36 ± 1.61^**^

Note: *p < 0.05 versus Con group, ^#^p < 0.05 versus Mb group, **p < 0.05 versus MSCs group n = 6.
