# Supplementary material for: Biological Membrane-Packed Mesenchymal Stem Cells Treat Acute Kidney Disease by Ameliorating Mitochondrial-Related Apoptosis
Source: Sci Rep. 2017 Jan 24;7:41136. doi: 10.1038/srep41136 (PMC5259718; doi:10.1038/srep41136)
Supplement: Supplementary Materials [file srep41136-s1.pdf]

# Supplement Information

# Biological Membrane-Packed Mesenchymal Stem Cells Treat Acute Kidney Disease by Ameliorating Mitochondrial-Related Apoptosis

Xiaodong Geng[1]*, Quan Hong[1]*, Weiwei Wang[2], Wei Zheng[1], Ou Li[1], Guangyan Cai[1], Xiangmei Chen[1], Di Wu[1]

[1]*Department of Nephrology, PLA General Hospital, Institute of Nephrology, Beijing Key Laboratory of Kidney Disease,State Key Laboratory of Kidney Diseases, National Clinical Research Center for Kidney Diseases, 28 Fuxing Road, Beijing 100853, China;* [2]*Department of Thoracic Surgery, Peking Union Medical College Hospital, Chinese Academy of Medical Sciences and Peking Union Medical College, No. 1 Shuai Fu Yuan, Eastern District, Beijing 100730, China.*Xiaodong Geng and Quan Hong contributed equally to this work.*

**Supplementary Figure 1.** Akt, p-Akt, Bad, Bcl-xl, Cytochrome c, cleaved caspase-9 and cleaved caspase-3 protein expression.

From left to right: Sham group, RM+BM group, RM+BM+MSCs group.

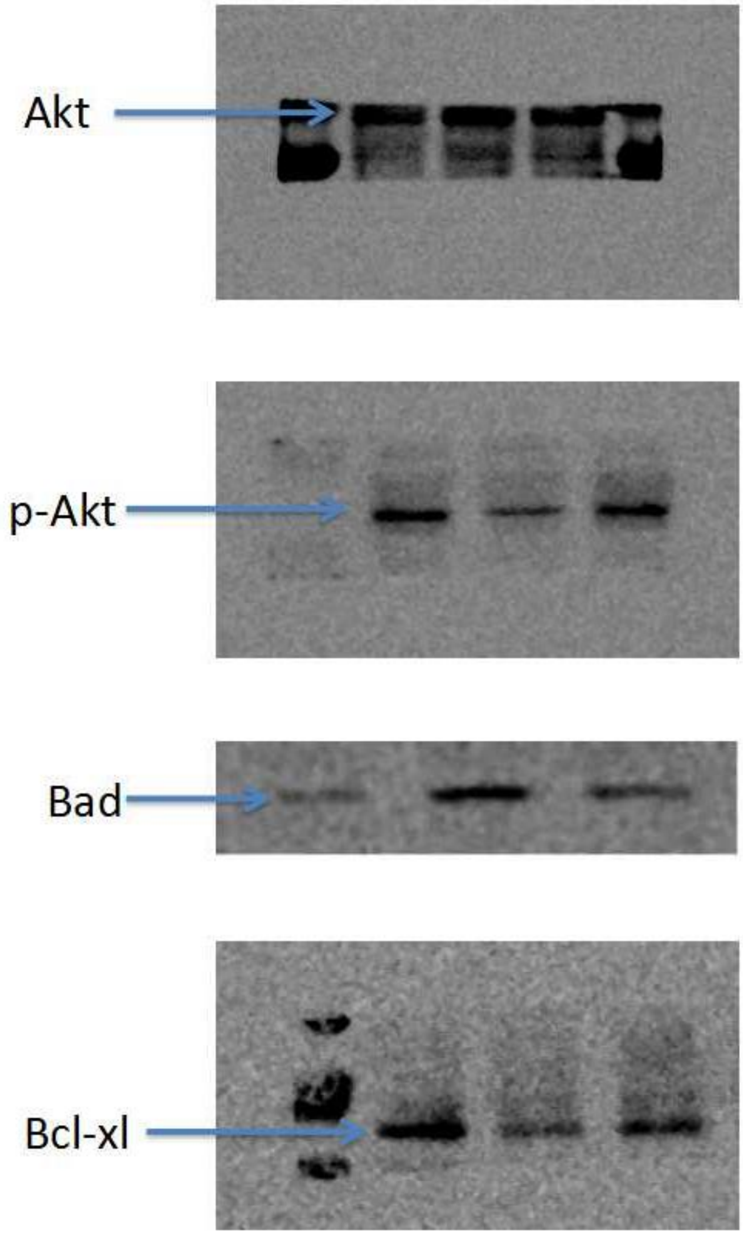

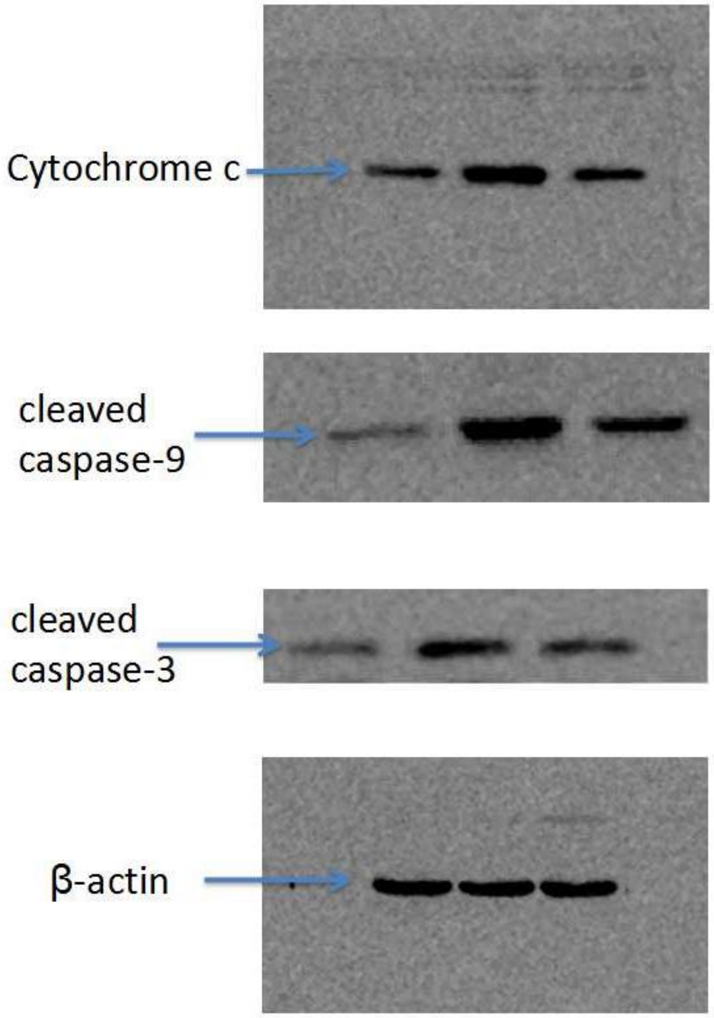
Cytochrome c
cleaved caspase-9
cleaved caspase-3
β-actin

**Supplementary Figure 2.** Effect of MSCs and LY294002 on the cell viability, cell injury, protein expressions.

From left to right: Control group, Mb group, Mb+MSCs group, Mb+MSCs+LY294002 group.

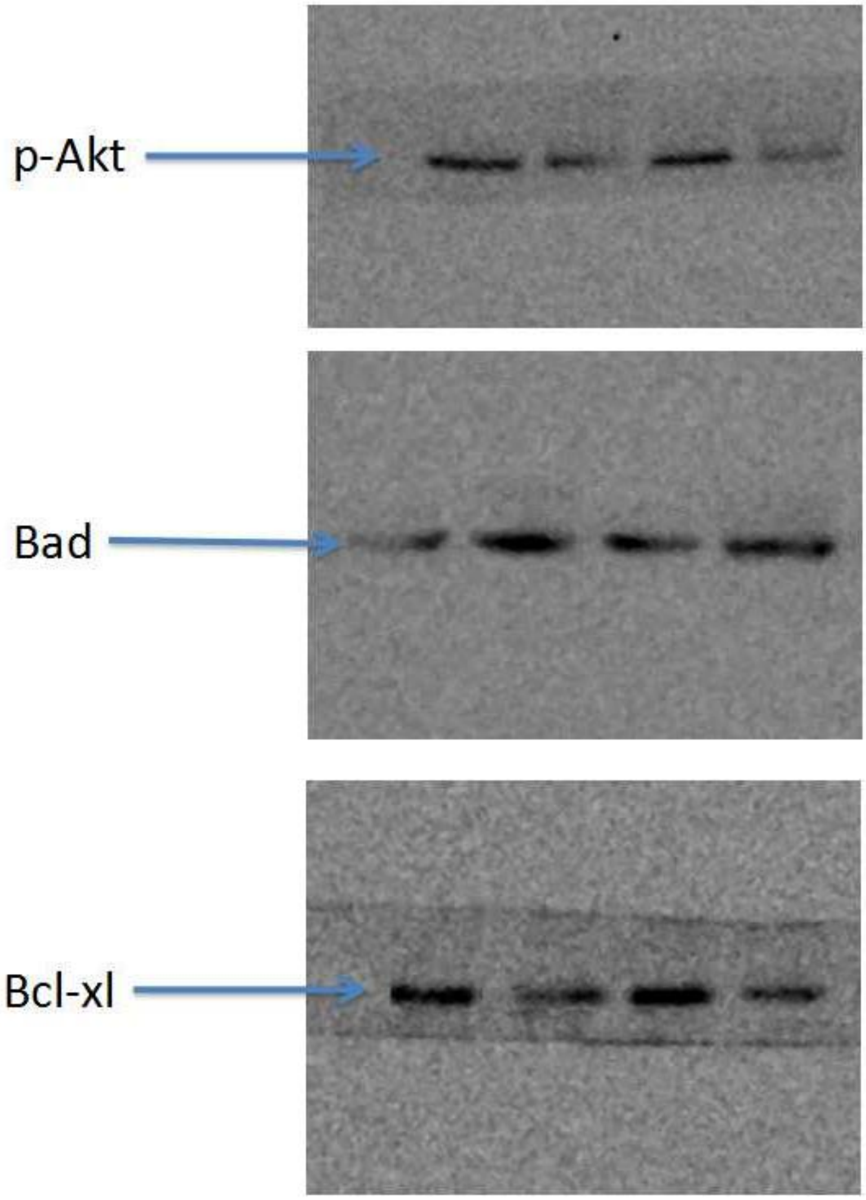

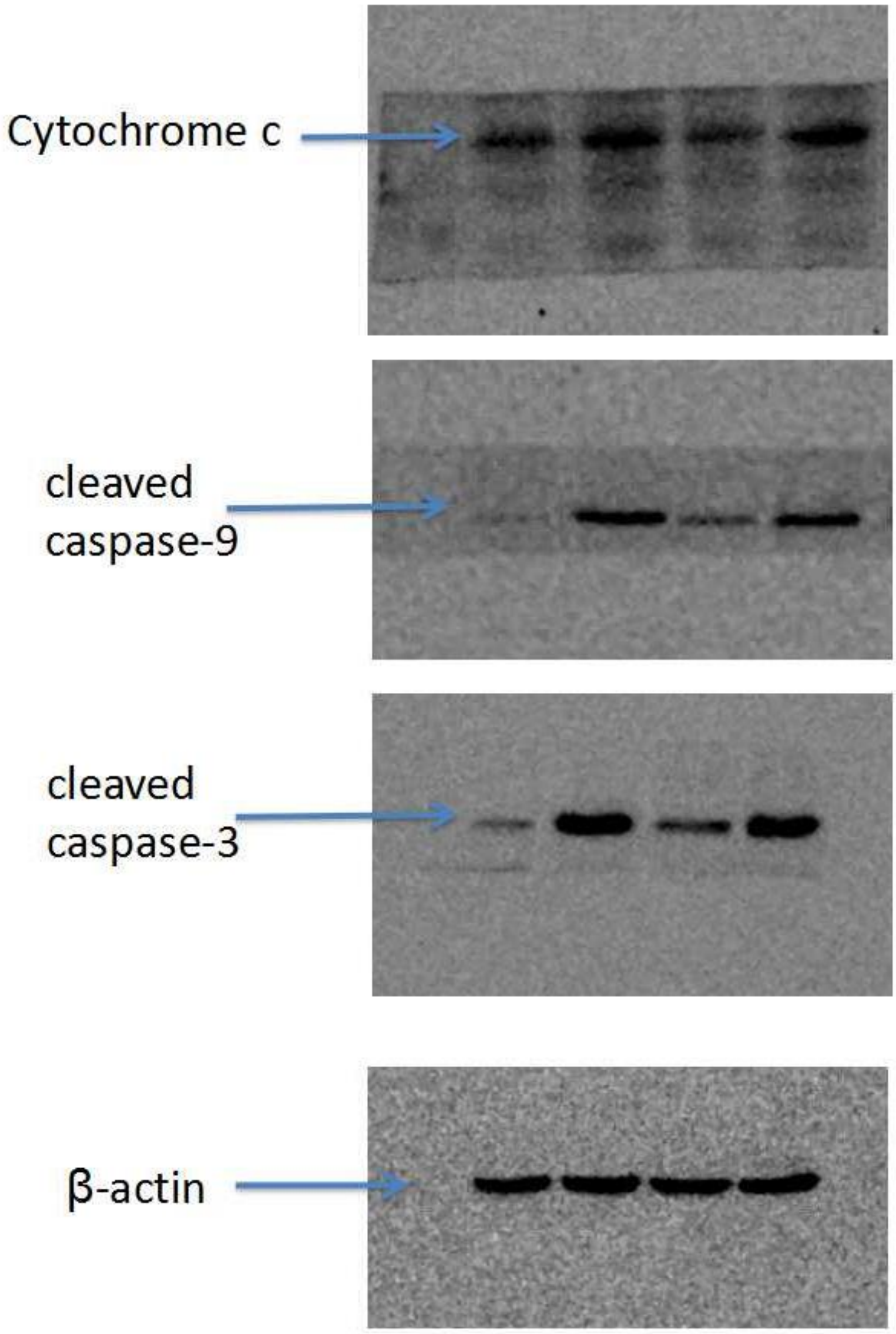
Cytochrome c
cleaved
caspase-9
cleaved
caspase-3
β-actin

**Supplementary Figure 3.** Effect of LY294002 on Akt phosphorylation and renal expression of mitochondria apoptotic proteinsin the kidneys of glycerol-induced AKI with MSCs treatment.

From left to right: RM+BM+MSCs group, RM+BM+MSCs+LY294002 group.

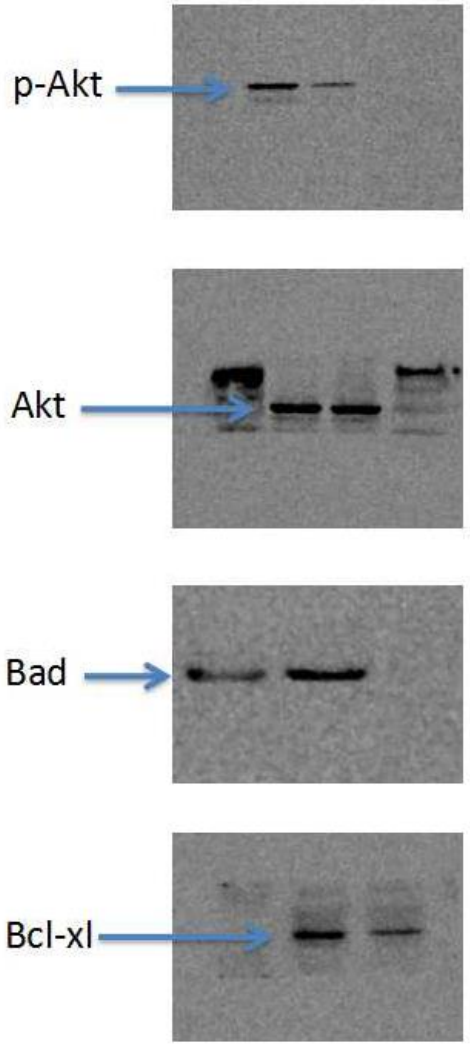

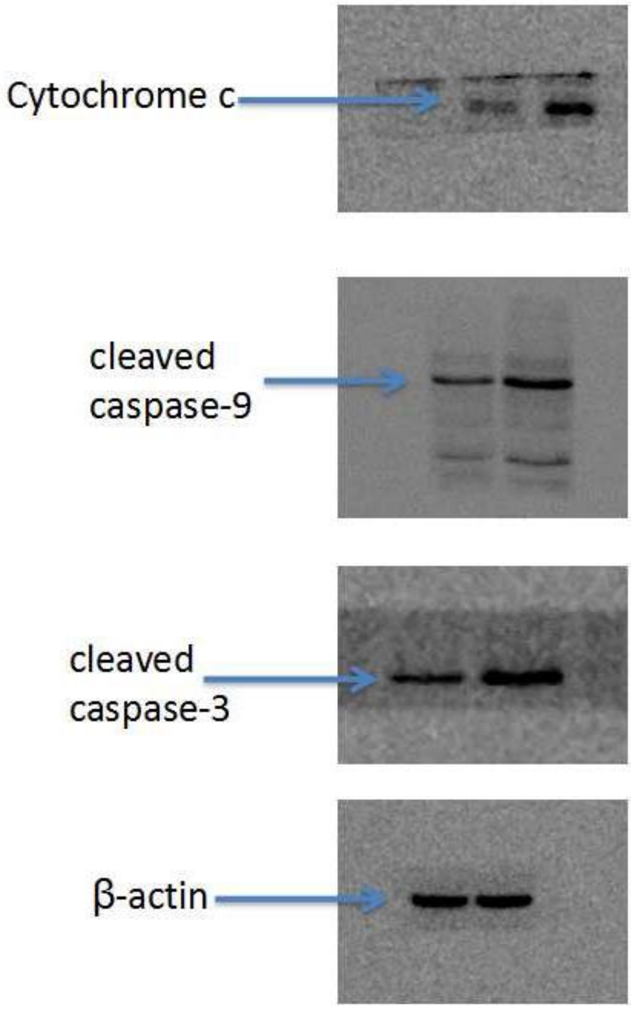


**Supplementary Figure 4.** Additional figures of immunofluorescencestaining of E-cadherin. (a) Sham group; (b) RM(rhabdomyolysis) group; (c) RM+BM(biological membrane) group; (d) RM+BM+MSCs (biological membrane-packed mesenchymal stem cells on the renal tissue) group; (e) Quantitative analysis of staining intensity showed a higher expression of E-cadherin in RM+BM+MSCs group compared with RM and RM+BM groups. Note:*p<0.05 versus the sham group, #p<0.05 versus the RM group, n=6.

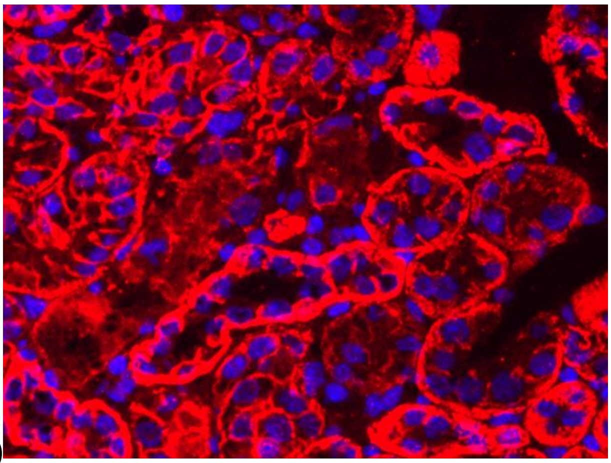
(a)

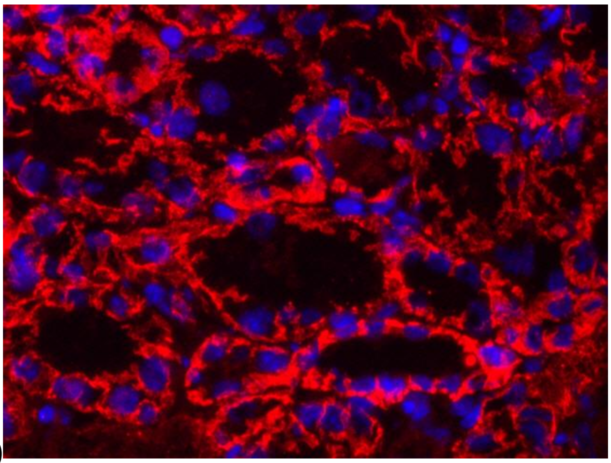
(b)

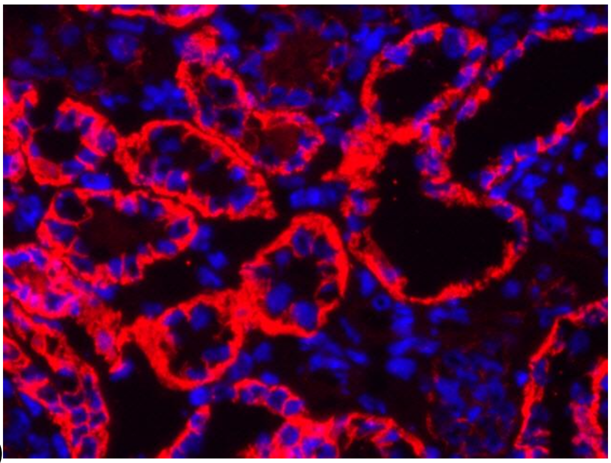
(c)

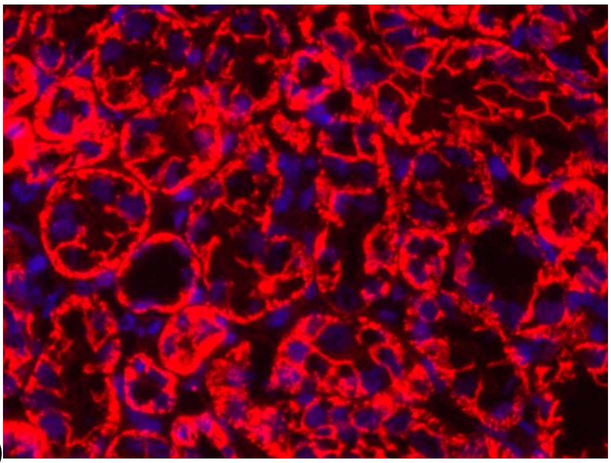

(d)

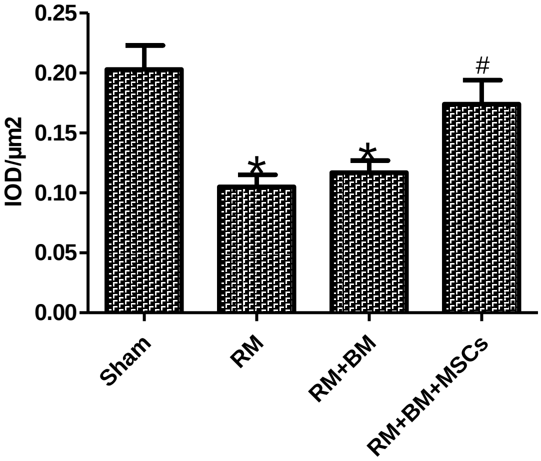


(e)
